# The Scottish RD survey 10 years on: the increasing incidence of retinal detachments

**DOI:** 10.1038/s41433-022-02123-1

**Published:** 2022-06-01

**Authors:** Mariam El-Abiary, Fatemah Shams, Colin Goudie, David Yorston

**Affiliations:** 1 Tennent Institute of Ophthalmology, Gartnavel General Hospital, 1053 Great Western Road, Glasgow, G12 0YN UK; 2 Princess Alexandra Eye Pavilion, Chalmers Street, Edinburgh, EH3 9HA UK

**Keywords:** Health occupations, Epidemiology

## Abstract

**Background:**

The Scottish RD Survey reported an incidence of 12.05/100,000/yr in 2009. Data published from Denmark recently confirmed a 50% increase in RD presentations over the last 16 years. We set out to repeat the Scottish RD survey to determine if a similar trend has been observed in Scotland.

**Methods:**

All 16 Scottish VR surgeons, who make up the collaboration of Scottish VR Surgeons (SCVRs) were asked to prospectively record all primary RDs presenting from 12th August 2019 to 11th August 2020. For consistency, the case definitions were the same as for the 2009 Scottish RD Survey. Basic demographic and clinical features were recorded. Age specific incidence was calculated from mid-year population estimates for 2019 obtained from the National Records of Scotland.

**Results:**

There were 875 RRDs recorded, which gives an updated incidence of 16.02/100,000/year in Scotland. 62.8% occurred in males and the greatest increases were seen in males aged 50–59 (*p* = 0.0094), 60–69 (*p* = 0.0395) and females aged 40–49 (*p* = 0.0312) and 50–59 (*p* = 0.0024). The proportion of pseudophakic RRDs in this study is 29.4% (253/860). Compared to the 21.6% in the 2010 study, this represents a 28% increase (*χ*^*2*^ = 11.03, *p* = 0.0009). The proportion of macula-off RRDs remained generally stable at 58%.

**Conclusion:**

Our study confirms that RRD is becoming more common in the UK, reflecting almost identical findings from Denmark. This trend is in part due to increasing myopia, increasing pseudophakia, and possibly other factors. This should be considered when planning VR services and allocating resources in the future.

## Introduction

Rhegmatogenous Retinal Detachment (RRD) is a sight threatening condition which requires prompt surgical management in a specialist centre. In 2010, the Scottish Retinal Detachment Study reported an annual incidence of 12.05/100,000/year [1]. The highest incidence was in men aged 50–70. Other studies from Europe in the last decade have found incidence rates ranging between 7.65/100,000/year and 26.2/100,000/year [2–5] with an average annual incidence of 13.3/100,000/year [6].

Recognised risk factors for RRD include male gender, 50–70 age group, peripheral retinal degeneration, history of trauma, cataract surgery and myopia [7, 8]. Hospital episode statistics show a gradual increase in RRD in Scotland, from a reported incidence of 9.36/100,000/year in 1987 to 13.61/100,000/year in 2003 [9]. Informal discussions with Vitreoretinal surgeons in Scotland suggested that this trend has continued. A recent study from Denmark has reported an increase in RRD incidence by over 50% between 2000 and 2016, the equivalent of an increase of 0.4/100,000/year [10]. Van Leeuwen reported a similar increased incidence in the Netherlands [5].

Recently, data from Hospital Episode Statistics has been used to demonstrate an increase in the number of procedures carried out by NHS England for RRD [11]. However, this data may include more than one operation for a single RRD and does not distinguish between rhegmatogenous and other types of retinal detachment.

Not all the literature reports an increase in RRD incidence. Meta-analysis in a systematic review of RD incidence in Europe reported a slight increase from 11.5 to 13/100,000/year [6]. The authors concluded that the incidence of RRD was unchanged, but there was a high level of heterogeneity between the studies included.

The purpose of this study was to determine the current incidence of RRD in Scotland and compare it to the Scottish RD Study in 2007–9 [1]. By using the same methodology as the original study, we aimed to provide definitive evidence of any change in the annual incidence of rhegmatogenous retinal detachment. Scotland has a well-defined and relatively homogenous population, with all RD repairs taking place in one of the six vitreoretinal centres. Moreover, there is a collaborative national network of 16 SCottish VitreoRetinal Surgeons (SCVRS) who cover all 14 Scottish health boards. NHS Scotland provides a primary care service via community optometrists and both specialist retinal consultations, and surgery, are free of charge to all patients.

## Methods

All sixteen VR surgeons based in six VR units across Scotland were contacted with the scope of the study and provided with blank spreadsheets to collect anonymous data between 12th August 2019 and 11th August 2020. To ensure that our results were comparable with the original Scottish RD study, we used the same case definitions and methods [12]. Each of the surgeons was prompted to return their completed spreadsheet to a principal investigator (ME) every month. Where required, surgeons cross-referenced their data with the theatre logbooks to maximise data capture.

Patient demographic data (age and sex), and details of the presenting features of the RRD were recorded (visual acuity, number of clock hours detached, type of retinal break, lens status, macula status and surgery performed). Exclusion criteria included recurrent RRD, RRD associated with posterior penetrating injury (including previous vitrectomy), and combined tractional and rhegmatogenous RD. No patient identifiable data was collected; therefore, no ethical approval was required. Authorisation for this study was provided by the local Caldicott Guardian.

From the National Records of Scotland, there we obtained mid-year population estimates for 2019 [13]. Age-specific incidence was calculated by dividing the frequency of RRDs by the population of the corresponding age group at that time. Data was collated in Microsoft Excel and statistical analysis performed in MedCalc® Statistical Software version 20.006 (MedCalc Software Ltd, Ostend, Belgium; https://www.medcalc.org; 2021). The χ^2^ test was used to compare the incidence rates between both studies and a *p* value of <0.5 was considered significant.

## Results

### Incidence

During 12 months of data collection, there were 875 RRDs recorded, which gives an incidence of 16.02/100,000/year in Scotland.

### Age and sex distribution

**Fig. 1:**
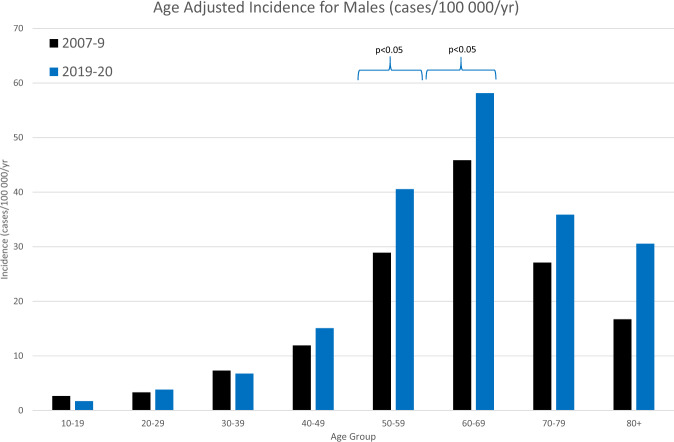
Age-Standardised Incidence rates for males comparing 2007–9 study to current study. Significant increase in incidence seen in 50–59 and 60–69 age groups.

**Fig. 2:**
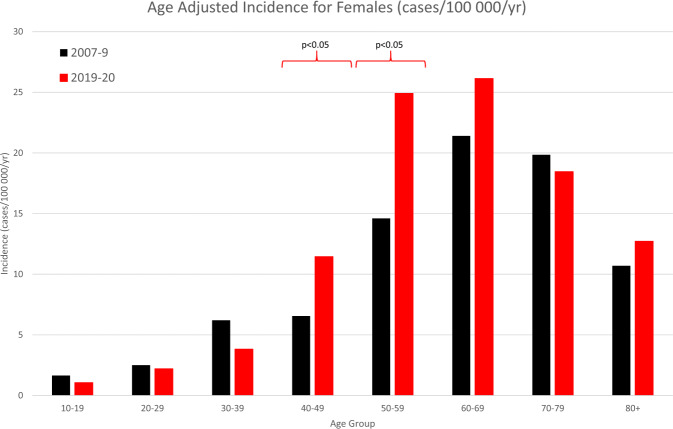
Age-Standardised Incidence rates for females comparing 2007–09 study to current study. Significant increase in incidence seen in 40–49 and 50–59 age groups.

The total incidence has increased from 14.75 to 20.39 (*p* < 0.0001) in males and 8.74 to 11.5 (*p* = 0.0012) compared to 2010. Table 1 summarises the baseline characteristics of the RRDs in this study period compared to the 2010 study. Figures 1, 2 show the incidence rates in each age group for males and females respectively, as compared to the 2010 study. The greatest increases were seen in males aged 50–59 (*p* = 0.0094) and 60–69 (*p* = 0.0395) and females aged 40–49 (*p* = 0.0312) and 50–59 (*p* = 0.0024). Unsurprisingly, this has resulted in a different age distribution, with a higher percentage of retinal detachments in the 50–59 age group.

**Table 1:** Baseline characteristics of the study population in comparison with 2007–9 study.

	2007–2009		2019–2020	
	24 months		12 months	
Total RRD Count	1202		875	
	No of eyes	%	No of eyes	%
Sex				
Male	735	61	543	63
Female	467	39	322	37
Age group				
<10	2	0.2	0	0
10–19	27	2.2	9	1
20–29	40	3.3	22	2.5
30–39	90	7.4	39	4.5
40–49	145	12	90	10
50–59	292	24	259	30
60–69	371	31	269	31
70–79	179	15	127	15
>80	56	4.7	54	6.4
Laterality				
Right	661	55	439	51
Left	522	43	417	48
Both	18	1.5	–	–
Lens status				
Phakic	920	77	601	70
Pseudophakic	260	22	253	29
Aphakic	22	1.8	6	0.7
With PVD				
U tear	975	86	739	84
GRT	15	1.3	14	1.6
Without PVD				
Round Hole	56	4.9	62	7.1
Dialysis	67	5.9	28	3.2
Schisis	17	1.5	9	1
Quadrants				
1	247	23	144	17
2	516	49	441	52
3	211	20	167	20
4	87	8.2	100	12
Macula status				
On	480	43	359	42
Off	650	58	498	58

### Lens status

The proportion of pseudophakic RRDs in this study is 29.4% (253/860). Compared to the 21.6% in the 2010 study, this represents a 28% increase (*x*^2^ = 11.03, *p* = 0.0009).

### Extent of detachment

The percentage of macula-off RRDs remains stable at 58% (495/854) when compared to the previous study. There was a reduction in the proportion of detachments limited to one quadrant, and an increase in the percentage of total detachments. These findings suggest it is unlikely that the higher incidence of RD is due to better case ascertainment or earlier diagnosis.

## Discussion

### New incidence of RRD in Scotland

This study revisits the 2010 census by Mitry et al which reported an RRD incidence of 12.05/100,000/year [1]. We estimate the current incidence of RRD is 16.02/100,000/year. Compared to the 2010 study, this represents an incidence ratio of 1:1.315 (95% CI 1.18–1.46, *p* < 0.0001). In both the original study and this study, there were 16 vitreoretinal surgeons based in six specialist centres across Scotland, suggesting that the provision of RRD surgery remained consistent in both time periods.

This is similar to data from Europe, where incidence ranges from 13.7/100,000/year to 26.2/100,000/year [2–5]. The methodology utilised in these reports varies. Most use either hospital episodes, or admissions (which depend on accurate coding of procedures); national registries or databases (which are not universally available); or reporting from a collaboration of VR surgeons, usually in smaller cohorts. Our study used this last method, which includes all RRD whether or not they have been treated. In our cohort, there were only three eyes that did not have surgery.

The RRD incidence in Scotland is higher than the incidence in Europe reported by Li in a meta-analysis which examined 5 European studies from 1999 to 2014 and found a pooled mean incidence of 13.3/100,000/year [6].

### Effect of SARS-CoV-19

During our prospective data collection period, it was noted that the SARS-CoV-19 pandemic and the associated lockdown measures had a significant impact on RD presentations [14–16]. The official dates of lockdown in Scotland were 24th March 2020 to 29th May 2020, and during this time there was a 53% decrease in the number of RD presentations in Scotland. Excluding the data from this lockdown period (65 days), there were 775 RRD presentations in the remaining 300 days of the study period. If we extrapolate this value, the incidence would be 942 or 17.24/100,000/year. This represents a 41% increase from 2007–9 (incidence ratio is 1:1.483 (95% CI 1.28–1.57, *p* < 0.0001)). We believe that this is a more accurate estimate of the true incidence of RRD in Scotland

### Increasing Incidence of RRD

In Scotland, an analysis of hospital episodes due to RRD between 1987 and 2006 showed an increase of 1.9% per year over the 20-year period [9]. Similar to our results, the greatest increase was observed in men in the 40–59 age group. In England, hospital admissions for retinal detachments were generally stable from the 1960s but increased significantly from 1999 onwards [11]. Episodes involving RRD increased from 7/100,000 in 2000 to 19.7/100,000 in 2018 and the highest rates were again seen in the males ages 65–74 age group [17]. Although our study uses a different methodology, it confirms increasing RRD incidence.

Increasing incidence has also been reported in Denmark [2, 10], France [18], the Netherlands [5], and Croatia [4]. Between 2000–2016, the age- and sex-standardised RRD incidence in Denmark increased by more than 50% for both men and women, but the largest increase was seen in men aged over 50 [10]. Recently, data from the Netherlands revealed a 44% rise in RRD incidence in the last seven years. Once again, the greatest increase was observed in males ages 50–70 [5]. A nationwide database study conducted in France confirmed a 12.63% rise in annual RRD incidence between 2010 and 2015, with almost twice as many males as females affected in the 60–74 age group [18].

The increase in RRD incidence is likely to be due to more cataract surgery, and a higher prevalence of myopia.

Cataract surgery increases the risk of RRD [19, 20]. Using the non-operated fellow eye as a reference, phacoemulsification is associated with a 4-fold increase in RRD regardless of patient age or gender [21]. This risk is highest in the first 6-month post-operative period, plateaus at two years, but remains considerably higher than the non-operated eye for up to 10 years.

In the 2010 Scottish RD study [1], 21.8% of recorded RRDs were pseudophakic. In this study, the proportion has increased to 30%. This is comparable to other recent studies from Europe, where the percentage of pseudophakic RRD varies between 15.96% [4], 33.5% [3], and 37.4% [5, 22]. In terms of absolute numbers, there were 141 pseudophakic patients in the original study from 2007–09, compared to 253 in the current study. However, the total number of RRDs has increased by over 250, which suggests that larger numbers of cataract operations does not fully explain the increase in RRD incidence. A recent retrospective database review by the British and Eire Association of Vitreoretinal Surgeons (BEAVRS) reported that pseudophakic RRD has a significantly higher male predominance compared to phakic RRDs, which also supports the role of pseudophakia in increasing RRDs [23]. Van Leeuwen also observed an increase in both phakic and pseudophakic RRDs suggesting that increased cataract surgery alone could not account for the rise in RRD [5]. According to Public Health Scotland (PHS), the number of cataract procedures performed in Scotland have increased from 42,676 in 2015/16 to 45,865 in 2019/20 [24]. This represents an average annual increase of 1.9%. The average annual increase in the incidence of RRD between 2009 and 2020 was 4.4%.

The biggest increase in RRD incidence is seen in males aged >50 in our cohort. The peak in the 50–69 age group likely corresponds to the age at which posterior vitreous detachment is most likely to occur. Other authors have reported that an increasing number of RD are treated by vitrectomy and internal tamponade [25], and this has been ascribed to reduced familiarity with scleral buckling techniques. However, our data offers another explanation–a higher proportion of RD are occurring in eyes that are likely to have a complete posterior vitreous separation.

An eye with spherical equivalent of −1.00D to −3.00D has a fourfold increase in RRD compared to an emmetropic eye, and this risk increases to tenfold if myopia exceeds −3.00D [8]. The prevalence of myopia is also increasing. Worldwide, an estimated pooled prevalence of 26.5% has been reported from a recent systematic review with a particular aggregation in South East Asia [26]. However, myopia is also becoming more prevalent in Europe [27, 28].

Although it is likely that a higher prevalence of myopia is contributing to the rise in RRD incidence, we cannot be certain, as we did not collect data on axial length, refractive error, or socio-economic status, which has been associated with myopia and RRD previously [1].

### Limitations

The strengths of this study include it’s prospective nature, and the inclusion of all incident RRDs in a well-defined and large population where the provision of the VR Surgery service remained consistent in the last 10 years.

We are confident that these figures are an accurate representation of the RRDs presenting to the Scottish NHS in this period due to the close collaboration of all Scottish VR surgeons. If this count is an underestimate, either due to poor data capture or due to the effects of the COVID-19 pandemic, there is a still a significant increase in the incidence of RRDs compared to 10 years ago, which indicates that the true figure is probably even higher. A weakness of this study is the limited clinical data (such as refractive status) collected, so we are unable to comment on the effect of myopia as a factor in the rising incidence of RRD.

## Conclusion

Our study shows that the incidence of retinal detachment is increasing in the UK. This confirms the findings of other studies from Europe. The rising incidence is likely to be caused by increasing cataract surgery, increasing myopia and possibly other undetermined factors. This increasing incidence should be considered when planning VR services in the future.

## Summary

### What was known before

- Rhegmatogenous Retinal Detachment is a blinding condition that require prompt management in a specialist centre.
- Ten years ago, the incidence of retinal detachments was 12.05/100,000/yr.
- A report from Europe found an increase in the incidence of RD in the last 16 years.

### What this study adds

- There is a statistically significant increase in the incidence of rhegmatogenous retinal detachments in Scotland in the last 10 years.
- This trend should be considered when planning for VR services in the future.

## Data Availability

The dataset analysed during the current study are available from the corresponding author on reasonable request. It is summarised in the published article in Table 1.
